# Robot‐assisted pyeloplasty for ureteropelvic junction obstruction complicating horseshoe kidney

**DOI:** 10.1002/iju5.12774

**Published:** 2024-09-02

**Authors:** Toshinori Nishikimi, Tomoyoshi Ohashi, Hiroshi Yamada, Hiroko Morikami, Yuriko Nagasaka, Hideki Mizuno

**Affiliations:** ^1^ Department of Urology Japanese Red Cross Aichi Medical Center Nagoya Daini Hospital Nagoya Aichi Japan

**Keywords:** horseshoe kidney, hydronephrosis, obstruction, pyeloplasty, robot‐assisted surgery

## Abstract

**Introduction:**

Horseshoe kidney is characterized by midline fusion of the lower poles of both kidneys. We report a case where robot‐assisted pyeloplasty was performed for hydronephrosis complicating horseshoe kidney.

**Case presentation:**

A 31‐year‐old man repeatedly developed fever since childhood. He visited a local clinic for fever and left back pain, where he was diagnosed and treated for acute pyelonephritis. Abdominal computed tomography led to a diagnosis of horseshoe kidney and associated left ureteropelvic junction obstruction. He was referred to our hospital for pyeloplasty because of persistent left back pain. Robot‐assisted left pyeloplasty was performed using the Anderson–Hynes technique with four ports because crossing vessels were discovered intraoperatively. Isthmusectomy was not performed. The postoperative course was favorable. Computed tomography performed approximately 2 years post‐surgery showed improvement in hydronephrosis. No left back pain was reported.

**Conclusion:**

Our case experience suggests the utility of robot‐assisted pyeloplasty for patients with horseshoe kidney.

**Abbreviations & Acronyms**
CT: computed tomography
ESWL: extracorporeal shock wave lithotripsy

**Keynote message**

Horseshoe is associated with complications such as hydronephrosis and vesicoureteral reflux, often necessitating surgical treatment. Robot‐assisted surgery is frequently performed because it provides excellent maneuverability and favorable visualization. Herein, we report a case in which robot‐assisted pyeloplasty was performed for hydronephrosis complicating horseshoe kidney.

## Introduction

Horseshoe kidney is characterized by midline fusion of the lower poles of both kidneys, resembling a horseshoe. It is associated with complications such as hydronephrosis and vesicoureteral reflux, often necessitating surgical treatment. Robot‐assisted surgery is frequently performed because it provides excellent maneuverability and favorable visualization. Herein, we report a case in which robot‐assisted pyeloplasty was performed for hydronephrosis complicating horseshoe kidney.

## Case presentation

### History of presenting illness

A 31‐year‐old man (height: 181 cm; weight: 110 kg) presented with a chief complaint of fever and left back pain. He had a history of recurrent pyelonephritis. The patient had experienced repeated episodes of fever since childhood. He recently visited a local clinic with chief concerns of fever and left back pain, where he was diagnosed and treated for acute pyelonephritis. Subsequently, imaging studies including abdominal CT led to a diagnosis of horseshoe kidney and associated obstruction of the left ureteropelvic junction. Owing to persistent left back pain and his request for surgical treatment, the patient was referred to our hospital for pyeloplasty.

**Fig. 1:**
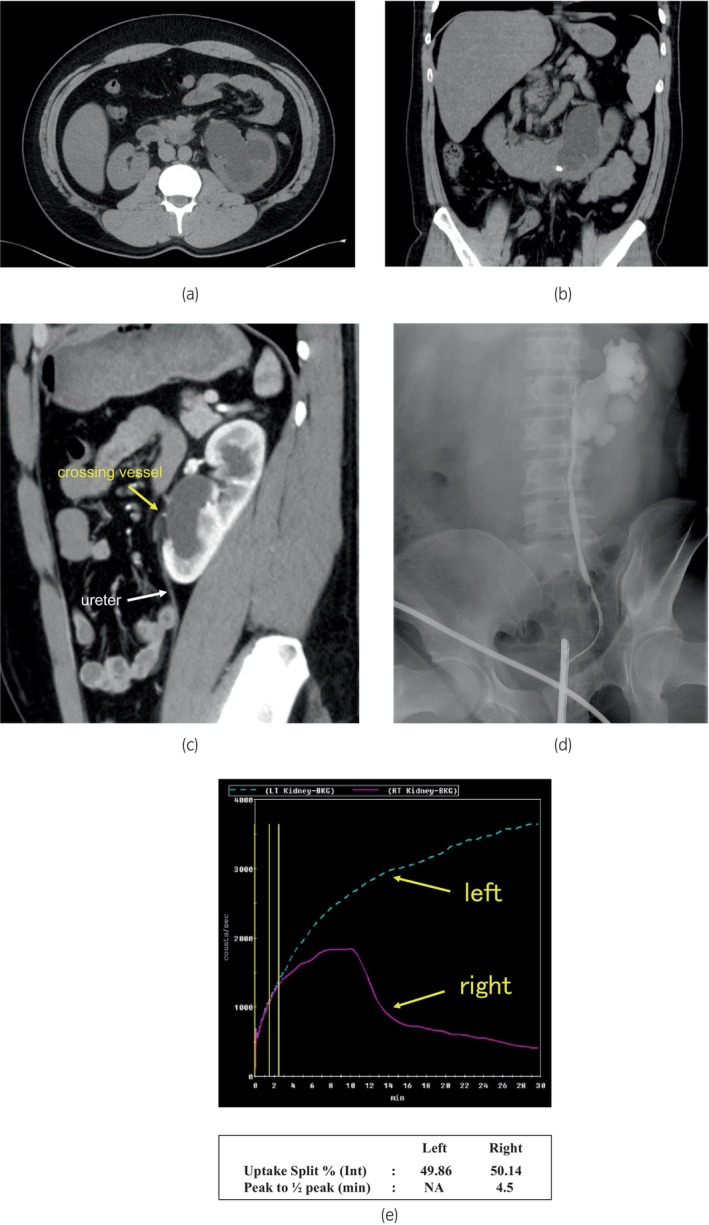
Imaging findings. *Preoperative computed tomography*: The left renal pelvis was dilated (a), and the lower poles of the kidneys were fused on the ventral side of the abdominal aorta to form horseshoe kidney (b). In addition, sagittal scans showed crossing vessels that appeared to be the cause of obstruction (c). *Retrograde pyelography*: It was unclear whether the urinary tract was displaced by the kidneys on retrograde pyelography (d). *Technetium‐99m‐mercaptoacetyltriglycine diuretic renography*: Diuretic renography performed using technetium‐99m‐mercaptoacetyltriglycine (99mTc‐MAG3) showing substantially delayed left renal excretion (e).

The creatinine level was 1.09 mg/dL, and the estimated glomerular filtration rate was 66.6 mL/min/1.73 m^2^. The results of hematologic tests including blood counts, biochemical tests, and coagulation tests were normal. Abdominal CT revealed dilation of the left renal pelvis (Fig. 1a); the lower poles of the kidneys appeared fused on the ventral side of the abdominal aorta to form a horseshoe kidney (Fig. 1b). Sagittal CT showed crossing vessels that appeared to be the cause of obstruction (Fig. 1c). Whether the urinary tract was displaced by the kidneys was unclear on retrograde pyelography (Fig. 1d). Technetium‐99m‐mercaptoacetyltriglycine diuretic renography showed substantially delayed left renal excretion (Fig. 1e). Diuretic renography was not performed preoperatively.

### Treatment course

The patient was diagnosed with horseshoe kidney complicated by ureteropelvic junction obstruction due to crossing vessels. Preoperative imaging did not depict displacement of the urinary tract due to horseshoe kidney. However, we decided to perform robot‐assisted pyeloplasty assuming that isthmus division would have to be performed in case urinary tract compression was detected intraoperatively. There appeared to be a small stone on CT. The stone was small and located deep, proximal to the isthmus, so it was not removed simultaneously during this surgery. We planned to perform ESWL at a later date. A successful procedure was defined as reduction in hydronephrosis and absence of symptoms.

### Intraoperative findings

**Fig. 2:**
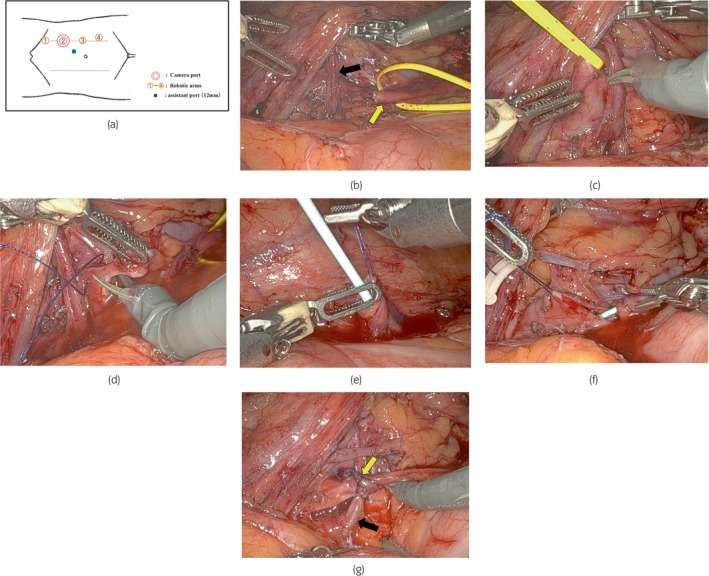
Surgical findings. *Port sites*: Four robotic arms were docked to the ports. The port for the left robotic arm at the most cranial side was placed at the edge of the rib on the lateral border of the rectus abdominis muscle. The camera port was placed on the lateral border of the rectus abdominis muscle at a distance of approximately 3–4 fingers' breadth caudally from the port for the left robotic arm. The port for the right robotic arm was placed laterally from the rectus abdominis muscle at a distance of approximately 3–4 fingers' breadth from the camera port. For the assistant's use, one 12‐mm port was placed between the port for the right robotic arm and camera port (a). *Surgical findings*: The lateral borders of the descending colon and spleen were incised. After the spleen and the pancreas were mobilized, the ureter coursing along the gonadal vein (yellow arrow) was identified and detached cranially. Then, the crossing arteries (black arrows) that appeared to be the cause of hydronephrosis were identified (b). After the ureter around the obstructed site and the renal pelvis were circumferentially detached (c), the renal pelvis was incised near the ureteral junction (d). The grasped obstructed site was resected. The ureter was also transected at a site with a normal diameter, and a sufficiently long longitudinal incision was placed. Then, a 6‐Fr double‐J ureteral stent was placed from the caudal side (e). Next, pyeloplasty was performed using the Anderson–Hynes technique with 4–0 monofilament absorbable thread to transpose the urinary tract (yellow arrow) in front of the crossing vessels (black arrows) (f and g). Isthmus division was not performed because there were no findings indicating displacement of the urinary tract due to the horseshoe kidney. The operative time was 3 h 11 min. The console time was 2 h 44 min. The volume of blood loss was 10 mL.

Surgery was performed under general anesthesia with the patient in the right‐side‐down lateral position, with the body slightly rotated dorsally from the vertical axis. Surgical findings are shown in Figure 2.

### Pathological examination

Edema and infiltration of plasma cells and lymphocytes were observed in the interstitium at the resected ureteropelvic junction. No malignant features were noted.

### Postoperative course

**Fig. 3:**
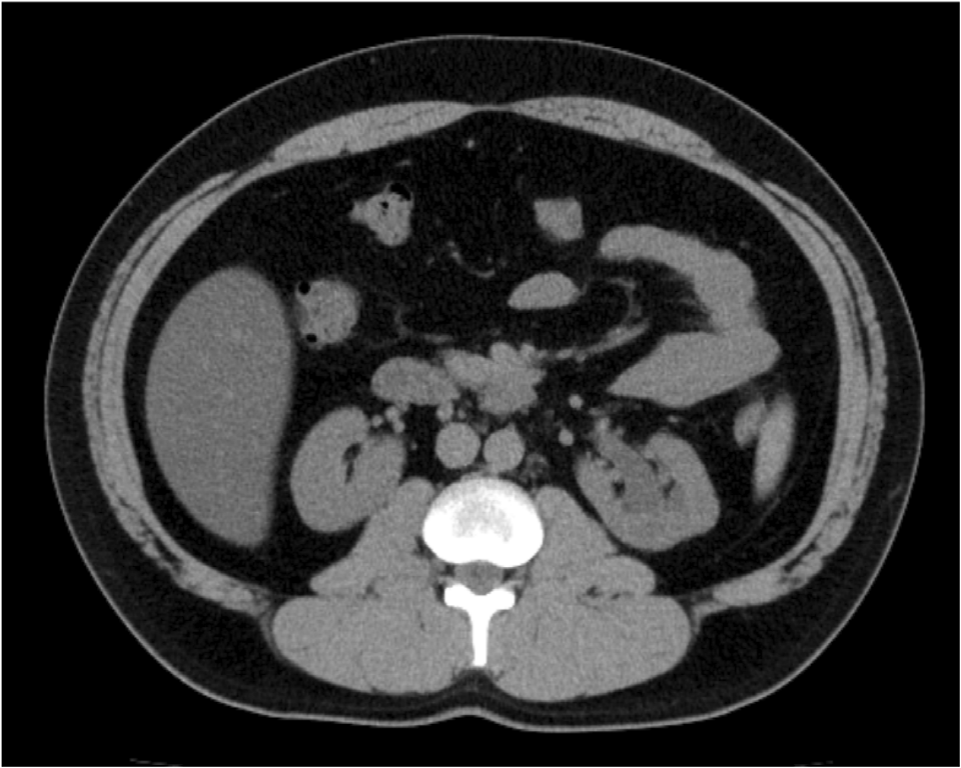
Postoperative computed tomography. Computed tomographic acquired approximately 2 years after surgery show improvement in hydronephrosis.

The postoperative course was favorable. The drain was removed on postoperative day 1, and the patient was discharged on postoperative day 2. Although removal of the double‐J ureteral stent was performed at the outpatient clinic on postoperative 3 months per the patient's wishes. Approximately 2 years after surgery, CT images showed improvement in the grade of hydronephrosis from grade 3 before surgery to grade 1 after surgery (Fig. 3). The patient's back pain disappeared, and no episodes of fever were noted thereafter. Although we recommended renography, the patient declined, preventing us from evaluating postoperative renal excretory function. A creatinine level of 0.89 mg/dL and an estimated glomerular filtration rate of 82.3 mL/min/1.73 m^2^ indicated an improvement in renal function.

## Discussion

Horseshoe kidney is congenital renal dysplasia, accounting for approximately 90% of renal fusion anomalies. Urinary tract infection or urolithiasis may occur when horseshoe kidney is complicated by vesicoureteral reflux or ureteropelvic junction obstruction. Aggressive surgical treatment is considered necessary in cases with renal impairment or pain associated with recurrent urinary tract infection or hydronephrosis.^1^ Studies have reported that one third of patients with horseshoe kidney have ureteropelvic junction obstruction.^2^ The other causes of ureteropelvic junction obstruction include compression by crossing vessels, high insertion of the ureter, intrinsic obstruction, and ureteral compression by displaced kidneys.^3^ Evidently, many variant blood vessels around the horseshoe kidney warrant attention.^4^ Pyeloplasty for comorbid ureteropelvic junction obstruction is a standard procedure and is most commonly performed using Anderson–Hynes technique, a type of dismembered pyeloplasty. Irrespective of the cause of obstruction, resection of the obstructed site, followed by reanastomosis of the renal pelvis and the ureter is safe, and yields favorable surgical outcomes. In recent years, conventional laparoscopic pyeloplasty^5^, ^6^ as well as robot‐assisted pyeloplasty^7^, ^8^, ^9^ have been performed. The advantages of robotic surgery include the use of articulated robotic arms that facilitate “detachment and incision through multidirectional approaches,” “fine sutures,” etc. The disadvantage is the high cost.^10^ When robot‐assisted pyeloplasty does not involve isthmus division, we believe that there are no major differences between “conventional pyeloplasty” and “pyeloplasty for horseshoe kidney” in terms of surgical procedures. However, because the anatomical features of horseshoe kidney differ from those of normal kidney in terms of the presence of an isthmus and the course of blood vessels, the advantages of robot‐assisted surgery, such as excellent visualization of surgical field by three‐dimensional imaging and ability to perform detachment and incision through multidirectional approaches, may be more pronounced for pyeloplasty for horseshoe kidney. Table 1 shows studies reporting robot‐assisted pyeloplasty for adults with horseshoe kidney. The transperitoneal approach to the surgical field is considered appropriate with respect to the anatomical positional relationship of horseshoe kidney and the objective to ensure sufficient space to maneuver the robotic surgical instruments; thus, the transperitoneal approach has been used in many studies.^7^, ^8^, ^9^ Meanwhile, whether isthmus division should be performed simultaneously for horseshoe kidney remains debatable. While one study reported a case requiring isthmus division,^9^ others achieved improvement with pyeloplasty alone.^7^, ^8^ Chammas *et al*.^7^ reported three cases of adult patients with horseshoe kidney who underwent robot‐assisted pyeloplasty. In all cases, isthmus division was not performed because of the absence of urinary tract compression by the horseshoe kidney, but renal function improved after 1 year of pyeloplasty alone. Similarly, Nishi *et al*.^11^ reported favorable outcomes with laparoscopic pyeloplasty in five patients with horseshoe kidney without isthmus division, because obstruction was caused by crossing vessels in all patients. Thus, we can infer that it is unnecessary to perform isthmus division for all patients, although it should be performed if preoperative imaging or intraoperative findings reveal displacement of the ureter by the horseshoe kidney. In our case, preoperative imaging did not show displacement of the urinary tract by the kidneys, although we had planned to perform isthmus division if intraoperatively displacement of the urinary tract by the horseshoe kidney would have been noted. Although isthmus division was not performed, the postoperative course was favorable.

**Table 1:** Studies on robot‐assisted pyeloplasty performed for adults with horseshoe kidney

First author	Publication year	Age/Sex	Affected side	Symptoms before surgery	Symptoms after surgery	Approach	Isthmus division	Crossed vessels	Operation time	Blood loss	Complications	Degree of hydronephrosis
Chammas *et al*.^7^	2006	25/M	Right	Lumbar pain	Asymptomatic	Transabdominal	No	Unknown	170 min	<100 ml	None	Normalized
		66/M	Right	Asymptomatic	Asymptomatic	Transabdominal	No	Unknown	125 min	<100 mL	None	Normalized
		43/F	Right	Lumbar pain	Asymptomatic	Transabdominal	No	Unknown	150 min	<100 ml	Urinary infection	Normalized
Spencer *et al*.^8^	2009	28/M 35/M	Right	Lumbar pain	Asymptomatic	Transabdominal	No	No	190 min	Minimal	None	Normalized
			Left	Lumbar pain	Asymptomatic	Transabdominal	No	No	90 min	Minimal	None	Normalized
Sheng *et al*.^9^	2016	47/F	Left	Lumbar pain	Unknown	Transabdominal	Done	Presence	123 min	<50 mL	None	Unknown
The current study	2024	31/M	Left	Lumbar pain	Asymptomatic	Transabdominal	No	Presence	191 min	10 mL	None	Improved

## Conclusion

We encountered a case where robot‐assisted pyeloplasty was performed for ureteropelvic junction obstruction complicating horseshoe kidney. Although isthmus division was not simultaneously performed because preoperative imaging showed no compression by the displaced kidneys, the clinical course was favorable.

## Author contributions

Toshinori Nishikimi: Conceptualization; writing – original draft; writing – review and editing. Tomoyoshi Ohashi: Conceptualization. Hiroshi Yamada: Conceptualization. Hiroko Morikami: Conceptualization. Yuriko Nagasaka: Conceptualization. Hideki Mizuno: Conceptualization.

## Conflict of interest

There are no conflicts of interest to declare.

## Approval of the research protocol by an Institutional Reviewer Board

The protocol for this research project has been approved by a suitably constituted Ethics Committee of the institution. The registration number is 001219.

## Informed consent

Informed consent was obtained from the subjects.

## Registry and the Registration No. of the study/trial

Not applicable.

## Ethical consideration

In consideration of ethical principles, the contents of this article comply with the regulations of the ethics committee of the Japanese Red Cross Aichi Medical Center Nagoya Daini Hospital.
